# The Follow-Up Necessity in Human Papilloma Virus-Positive vs. Human Papilloma Virus-Negative Oral Mucosal Lesions: A Retrospective Study

**DOI:** 10.3390/jcm13010058

**Published:** 2023-12-21

**Authors:** Armina Rushiti, Chiara Castellani, Alessia Cerrato, Marny Fedrigo, Luca Sbricoli, Eriberto Bressan, Annalisa Angelini, Christian Bacci

**Affiliations:** 1 Unit of Oral Pathology and Medicine and Odontostomatological Diagnostics, Section of Clinical Dentistry, Department of Neurosciences, University of Padova, 35122 Padova, Italy; arminarushiti@gmail.com (A.R.); cerrato.alessia92@gmail.com (A.C.); luca.sbricoli@unipd.it (L.S.); eriberto.bressan@unidp.it (E.B.); annalisa.angelini@unipd.it (A.A.); 2 Cardiovascular Pathology, University of Padova Medical School, University of Padova, 35122 Padova, Italy; chiara.castellani@unipd.it (C.C.); marny.fedrigo@unipd.it (M.F.)

**Keywords:** oral HPV, oral pathology, follow-up

## Abstract

Human papilloma virus (HPV) is known as the main cause of cervical cancer. Data also indicate its role in head–neck cancer, especially oropharyngeal cancer. The correlation between high-risk HPV and oral cancer is still controversial. HPV-related lesions of the oral cavity are frequent and, in most cases, benign. The primary aim of this study was to establish if there is a different follow-up necessity between HPV-positive compared to HPV-negative oral lesions. The secondary aim was to evaluate the recurrence of HPV-related lesions. All patients who underwent a surgical procedure of oral biopsy between 2018 and 2022, with ulterior histopathological examination and HPV typing, were examined. A total of 230 patients were included: 75 received traumatic fibroma as diagnosis, 131 HPV-related lesions, 9 proliferative verrucous leukoplakia, and 15 leukoplakia. The frequency and period of follow-up varied in relation to HPV positivity and diagnosis. This study confirms what has already been reported by other authors regarding the absence of recommendations of follow-up necessity in patients with oral mucosal lesions. However, the data demonstrate that there was a statistically significant difference in the sample analyzed regarding the follow-up of HPV-positive vs. HPV-negative patients. It also confirms the low recurrence frequency of HPV-related oral lesions.

## 1. Introduction

Human papilloma viruses or HPVs (an acronym for “human papilloma virus”) are non-enveloped DNA viruses belonging to the papillomaviridae family. They can infect not only humans but also different animal species in a species-specific way [1].

HPVs are epitheliotropic viruses that can cause a variety of lesions at cutaneous and mucosal sites; they can only replicate within epithelial cells. While on the skin, they are generally associated with the development of benign hyperplastic lesions (warts); for some HPVs with mucosal tropism, it has been widely demonstrated that they increase the risk of neoplastic transformation of the infected site [2]. Indeed, it has been estimated that high-risk HPV is present in 100% of cervical cancers worldwide [3]. In recent years, data on the association between HPV and oropharyngeal cancer have emerged, with an estimated 80% of oropharyngeal cancers in the United States and Western Europe being attributed to HPV [4].

Human papilloma viruses are divided into five genera, based on the analysis of their DNA: alpha-papillomavirus, beta-papillomavirus, gamma-papillomavirus, nu-papillomavirus, and mu-papillomavirus [5]. Due to their association with cancer, scientific research has mainly focused on the study of HPVs of the genus alpha. The biological mechanisms underlying neoplastic development are widely known [2]; the role of HPV16 in the development of cervical cancer has been demonstrated [3,6]. HPVs belonging to the genus alpha are classified according to the risk of malignant transformation of the infected site into low risk and high risk.

The HPV genome has about 8000 base pairs that code for seven or eight early viral proteins (E1, E2, E3, E4, E5, E6, E7, and E8), two late proteins (L1 and L2), and a “long control region” (LCR) [2]. Since the virus does not code for its own DNA polymerase, it needs the replicative mechanisms of the host cell in order to replicate. For this reason, viral replication occurs only in cells undergoing mitosis [7].

In a healthy epithelium, basal cells exit the cell cycle soon after migrating to the next layer, initiating a process of terminal differentiation. In the case of papilloma virus infection, E6 and E7 stimulate cell cycle progression, and the normal differentiation process is delayed [8]. Keratinocytes normally undergo terminal differentiation, but in the case of HPV infection, E7 stimulates the progression from G1-phase to S-phase of the cell cycle (the phase in which the cell’s genome replication occurs). The cell is stimulated to enter the S-phase even in the absence of mitogenic signals [9].

E7 associates with pRb, p107, and p130 proteins, tumor suppressors belonging to the retinoblastoma (Rb) family, which is involved in controlling cell cycle restriction. Since E7 alters their function, the cell is then stimulated to progress through the cell cycle [10]; E7 also causes accumulation of the tumor suppressor p16 in cells infected with HPV. For this reason, p16 is considered a marker of HPV infection in some head and neck cancers [11].

In a healthy epithelium, in case of uncontrolled cell proliferation, p53 induces cell apoptosis. p53 is a tumor suppressor with a dual function: it is involved in DNA repair or triggers apoptosis in cells with irreversible genome damage. Since E6 has the ability to induce the degradation of p53, keratinocytes infected with HPV are predisposed to accumulate gene mutations, which increase the possibility of malignant transformation. The ability to degrade p53, however, is a peculiarity of high-risk HPV and plays a key role in carcinogenesis. The role of E6 and E7 in the carcinogenesis process is evident in [6] and [12,13,14,15].

Studies in the literature report differing data on the prevalence of oral HPV in healthy subjects, with a median of 11% [16,17,18]. Several studies have tried to study the clearance of oral HPV infections, but the comparison is difficult due to the different follow-ups used by the various studies. Generally, the virus is completely eliminated over a period of 1–2 years [19]. However, in certain cases, high-risk HPV infection persists in basal layer cells (for about 10 years) and causes squamous cell carcinomas of the oropharynx [20].

Several authors have analyzed the possible association between HPV and squamous cell carcinoma of the oral cavity, as well as numerous oral potentially malignant disorders, including lichen planus, leukoplakia, and erythroplakia. A recent meta-review of the literature conducted on 52 studies, for a total of 2677 subjects with oral potentially malignant disorders of the oral cavity, reports a prevalence of oral HPV of 22.5%, significantly higher than the percentages reported for the healthy population [21]. In a systematic review of the literature published in 2011, it was reported that patients with oral lichen planus are five times more likely to have a HPV infection than healthy subjects [22]. In patients with leukoplakia, the prevalence of HPV ranges from 20.2% to 24.7% in the proliferative form [21]. Although the presence of HPV in leukoplasic lesions is frequent, several authors suggest that the data available are simply not enough to demonstrate the causal role of the virus in the development of leukoplakia nor the progression of this lesion to carcinoma [23].

In 2011, the IARC (International Agency for Research on Cancer) concluded that there was sufficient evidence for a causal role of HPV16 in oral cancer, while the role of HPV18 was considered possible [24].

A meta-analysis that included more than 2500 patients with oral cancer reports a prevalence of 23.5% of HPV-positive cancers, with a predominance of HPV16 (16% of cases) and HPV18 (8% of cases) [25]. A 2021 study reports that HPV16 infection may increase the risk of developing cancer in all oral cavity sites [26]. Lately, some authors have been recommending the use of narrow-band imaging for the early detection of oral squamous cell carcinoma, which showed high reliability, but it seems that oral lichen planus may lead to false positives [27].

A total of 82% of HPV-positive oropharyngeal carcinomas are due to HPV16 [28]. The prevalence of HPV infection in oral cancer is significantly lower than in oropharyngeal cancer. The reason for this different anatomical predisposition is not clear [29].

## 2. Materials and Methods

This study used a retrospective design. The primary aim of this study was to establish if there is a different follow-up necessity between HPV-positive compared to HPV-negative oral lesions. The secondary aim was to evaluate the recurrence of HPV-related lesions.

The population of this study was all the patients referred to the Dental Clinic of the University of Padova for the presence of lesions in the oral cavity from 1 January 2018 to 31 December 2022 who received an excisional or incisional biopsy.

The inclusion criteria were patients with the following clinical–histological diagnoses:

- Traumatic fibroma;
- HPV-related lesions (squamous papilloma, condyloma acuminatum, and verruca vulgaris);
- Proliferative verrucous leukoplakia (PVL);
- Leukoplakia.

Patients who received a clinical–histological diagnosis different from the above-mentioned ones were excluded.

Patients who did not respect the follow-up program were excluded.

Patients’ data were obtained from dental records. Age, sex, anatomical site of the lesion, positivity or negativity to the high-risk HPV group, positivity or negativity to the generic-risk HPV group, the frequency of follow-ups (e.g., follow-up at 4 months, 6 months, 12 months, etc.), and recurrence were recorded.

The molecular investigation for the search of HPV DNA was performed via a polymerase chain reaction (PCR) using a CE-IVD certified kit (Papilloma Virus Nested Kit, Experteam s.r.l., Venice, Italy) following the user manual by the Cardiovascular Pathological Anatomy Unit, followed by gel electrophoresis on agarose gel (Ultrapure Agarose, Thermo Fischer Scientific, Rome, Italy) and visualized using the fluorescence imaging system Alliance 2.7 (UVITEC, Eppendorf, Milan, Italy).

The genomes identified were divided into two groups, as follows:

1. Human papilloma virus generic-risk genotypes (HPV gr): 6, 11, 16, 18, 31, 33, 35, 39, 40, 42, 43, 45, 51, 52, 53, 54, 56, 58, 59, 66, 67, 68, 70, 71, 72, 73, 81, 82, 84, and 85;
2. Human papilloma virus high-risk genotypes (HPV hr): 16, 18, 31, 33, 35, 52, and 58.

Patients were identified with a code, and the data concerning them, collected during the study, were recorded, processed, and stored with this code only and not with the patient’s name, in accordance with EU regulation 2016/679, known as GDPR (General Data Protection Regulation), approved by the Institutional Review Board of Azienda Ospedaliera di Padova (CESC CODE 162n/AO/21 date 23 September 2021).

Informed consent was obtained from all subjects involved in the study.

A database was created using Microsoft Excel 2023©. Patients were inserted into the database in a completely anonymous form and were numbered with increasing prime numbers. The database was organized in a way that included some key items (Table 1).

The first entry corresponds to the patient’s code, entered anonymously. The second entry, “age”, indicates the age of the patient. The third entry, “sex”, indicates the sex of the patient (M/F). The fourth entry, “exam date”, indicates the date on which the bioptic examination was performed. The fifth entry, “diagnosis”, indicates the clinical–histological diagnosis, indicated with capital letters of the alphabet, and each letter represents a specific diagnosis; for example, the letter “A” indicates “traumatic fibroma of the oral mucosa”, the letter “B” indicates “HPV-related lesion”, the letter “C” indicates “proliferative verrucous leukoplakia (PVL)”, and the letter “D” indicates “leukoplakia” (Table 2).

The sixth entry, “anatomical site”, indicates the site where the sample was taken and in the database, it is marked with lowercase letters of the alphabet, and each letter represents a specific site; for example, the letter “a” indicates “lateral margin of the tongue”, the letter “b” indicates “retromolar trigon”, and so on, as can be seen in Table 3.

The seventh entry, “HPV hr”, indicates high-risk HPV positivity or negativity; positive cases were marked with 1, and negative cases were marked with 0. The eighth entry, “HPV gr”, indicates positivity or negativity to generic-risk HPV; positivity was indicated as 1, and negativity was indicated as 0. The ninth entry, “4-week follow-up”, indicates whether a follow-up visit at 4 weeks after the biopsy was performed. In case the visit took place, it was marked with 1; otherwise, it was marked with 0. The tenth entry, “2-month follow-up”, indicates whether there was a follow-up visit 2 months after the biopsy, and so on up to 5 years of follow-up. In case the visit took place, it was marked with 1; otherwise, it was marked with 0.

Possible cases of recurrence of HPV-positive lesions were also evaluated. Cases of recurrence were marked as “1r” during the various check-ups.

The data were analyzed using the statistical software R version 4.3.1 (R Foundation for Statistical Computing, Vienna, Austria). The R software studied the periodicity of follow-up frequencies in HPV-positive and HPV-negative patients. Dependency between qualitative and quantitative variables under evaluation was also tested using a Z-Test. A *p*-value lower than 0.05 was considered statistically significant.

## 3. Results

### 3.1. Sample Analysis

The total number of patients who underwent oral biopsy between 1 January 2018 and 31 December 2022 was 988, while the total number of samples on which HPV typing was performed was 284. However, not all of them respected the inclusion criteria; therefore, 230 cases were included in this study (as stated in Section 2).

**Figure 1:**
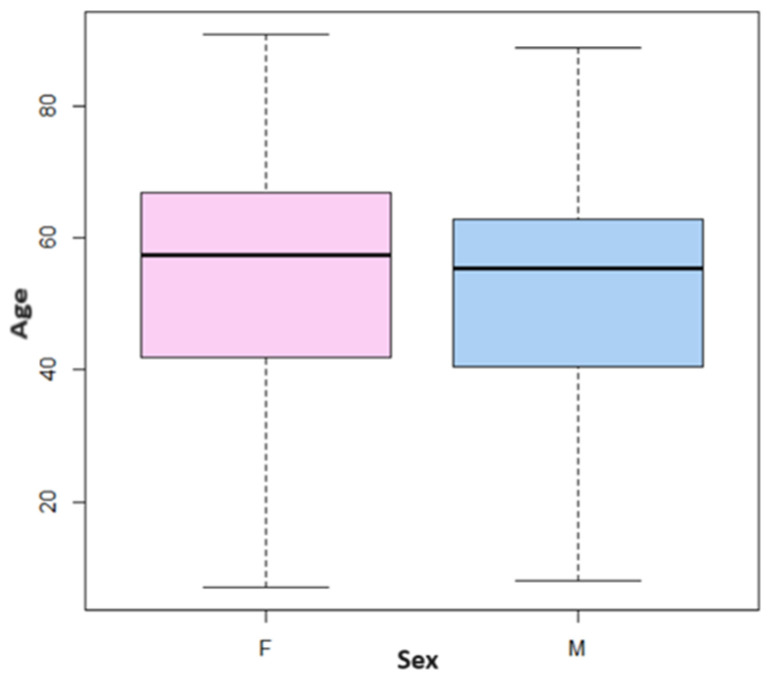
Age distribution for each sex.

The data showed that the patients were mostly over 50 (median age): for both sexes, the distributions are uniform and are concentrated between 42 and 67 years for women and between 40 and 63 for men (Figure 1).

**Figure 2:**
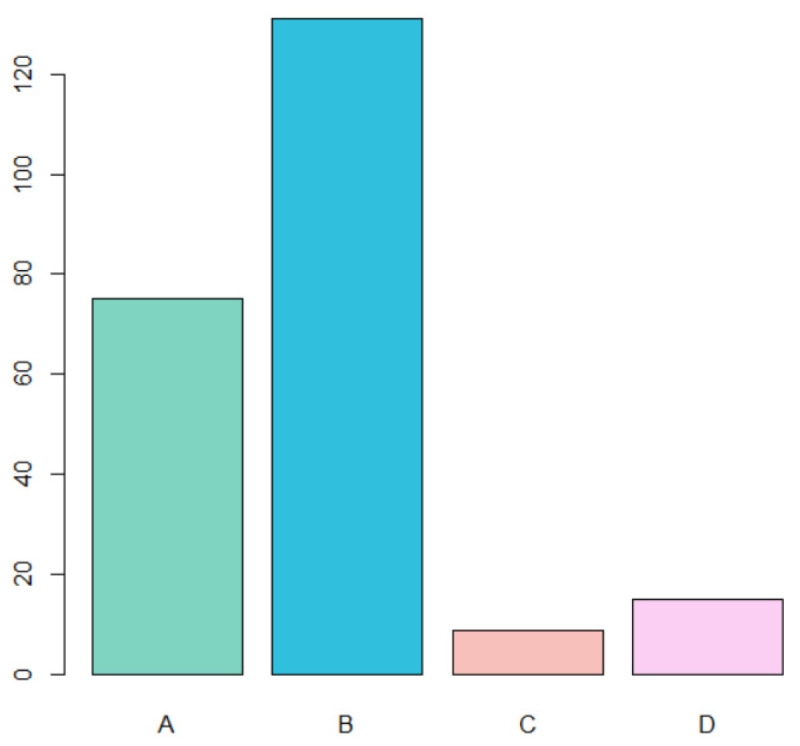
Distribution of each diagnosis type in the analyzed sample.

Of the 230 cases considered, the most frequent diagnosis was type B (HPV-related lesions). Type C (PVL) and D (leukoplakia) were much rarer. Specifically, (Figure 2):

- A total of 75 cases of traumatic fibroma (A) (32.6% of total cases);
- A total of 131 cases of HPV-related lesions (B) (56.9% of total cases);
- A total of 9 cases of PVL (C) (3.9% of total cases);
- A total of 15 cases of leukoplakia (D) (6.5% of total cases).

**Figure 3:**
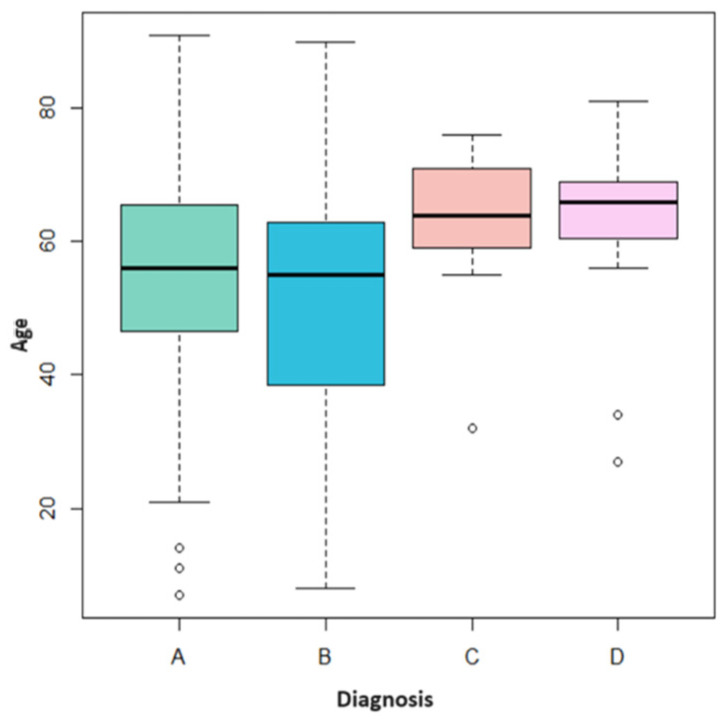
Age distribution by diagnosis: traumatic fibroid (**A**), HPV-related lesions (**B**), PVL (**C**), and leukoplakia (**D**).

Figure 3 shows how each type of diagnosis has a different age distribution. In the case of “traumatic fibroma” (A), the age distribution is uniform; the median is around 55 years, which is slightly higher than the median age of “HPV-related lesions” (B). In this case, however, the ages are less uniform, especially between the first and second quartiles, indicating greater variability in younger patients. Precisely, 12 patients under the age of 20 were diagnosed with “HPV-related lesions”. As for “PVL” (C) and “leukoplakia” (D), the distributions are much more concentrated on high values; both medians are positioned around 65 years of age with few younger outliers.

The outlier case with PVL was a 32-year-old patient, while the two outliers diagnosed with leukoplakia were 34 and 27 years old.

**Figure 4:**
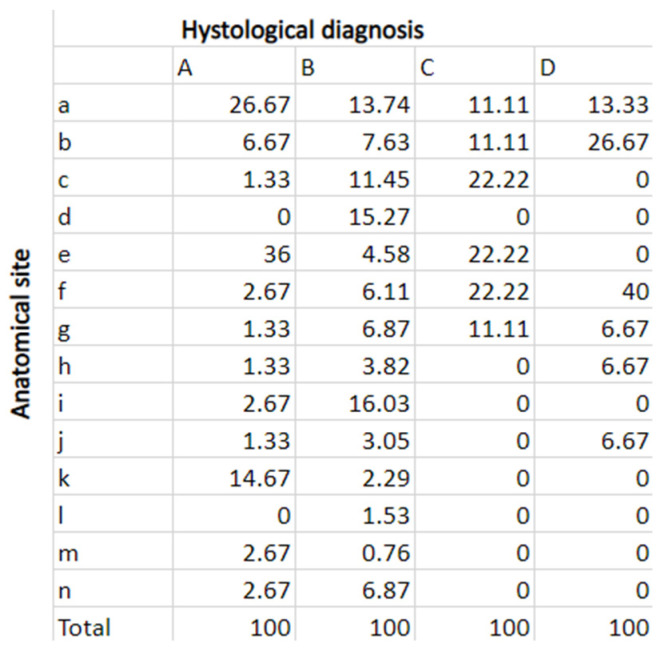
Distribution of the anatomical sites according to the diagnosis.

As can be seen from Figure 4, the most frequent anatomical sites for traumatic fibroma were, in order, buccal mucosa, lateral margin of the tongue, and labial retro-commissure.

Regarding HPV-related lesions, the most frequent sites were the dorsal surface of the tongue, the mucosa of the soft palate, and the lateral margin of the tongue.

In PVL patients, the most affected sites were the hard palate mucosa, vestibular gingiva of the upper maxillary, and buccal mucosa.

In patients with leukoplakia, the most frequent site was the retromolar trigone.

### 3.2. Follow-Up Comparison

Analyzing the comparison of follow-ups between HPV-positive lesions compared to HPV-negative lesions, it emerges that:

- Patients diagnosed with traumatic fibroma (A) negative for both HPV hr and HPV gr were seen only after 1 month for a check-up visit. Patients with positive HPV hr received a further check-up after 6 months;
- Patients diagnosed with HPV-related (B) lesions negative for both HPV hr and HPV gr were seen on average only after 1 month; those who tested positive for HPV gr were seen after 1 and 6 months. The patients positive for HPV hr typing were generally seen at 1, 6, and 12 months for check-ups;
- Patients diagnosed with PVL (C) received, on average, periodic follow-ups every 4 months in case of HPV positivity and follow-up every 6 months in case of HPV negativity;
- Patients diagnosed with leukoplakia (D) received periodic follow-ups every 6 months regardless of the positivity or negativity of HPV.

A multinomial model was built, in which the database was divided between the 80% and 20% rules, and then the error was calculated on the smallest set of tests. The output showed that there is a statistically significant difference between the number of follow-up visits (“sum” variable) in all four groups. Positive and negative HPV cases have a statistically significant difference in the number of follow-ups (*p* < 0.05) but are related to histological types A, B, and C. There were no statistically significant data regarding sex and age (*p* > 0.05).

### 3.3. Recurrence Rate of HPV-Related Lesions

Recurrence of HPV-related lesions occurred in 4.6% of cases; 50% relapsed at 12 months, 33.3% at 6 months, and 16.7% at 16 months. On average, relapses occurred within the first year (Table 4).

## 4. Discussion

### 4.1. Oral Lesions Follow-Up

No data similar to those reported in this study are available in the literature: there are no comparison articles available on a follow-up basis of patients presenting HPV-positive or HPV-negative lesions. However, numerous studies [30,31,32,33,34,35] and systematic reviews recommend a certain follow-up of some oral lesions, considering only the diagnosis.

A study that included 1566 samples compared the histological and clinical diagnosis of oral lesions [36]. The study showed that in 31.5% of cases, the dentists’ clinical diagnosis was wrong. Given the high error rate, good clinical practice should always consider the submission of excised samples for histologic examination, which is why we also decided to include recurrent benign lesions, such as traumatic fibromas [36].

There are no studies that report the follow-up necessity of traumatic fibromas, as surgical excision is resolutive and has a low recurrence rate [37,38]. For this reason, patients with traumatic fibromas were not included in a follow-up program but were seen after 4 weeks for a single check-up.

In a review published in 2021, Fiorillo et al. wanted to clarify the main features of HPV-related lesions of the oral cavity, the symptoms, the treatment, and the approach to be taken in HPV-positive patients. Given the high incidence rate of oropharyngeal carcinomas due to HPV (in particular HPV16) and given the still controversial correlation between HPV and oral cancer, the authors conclude not to underestimate HPV oral infections and suggest a multidisciplinary approach in the treatment plan and the follow-up program. Therefore, they propose a follow-up program carried out by different specialists (dentist, ENT, and gynecologist); however, they do not provide any information on the timing within which it should be carried out [34].

The data from the current study show a statistically significant difference in follow-up between patients diagnosed with HPV-related lesions with negative typing and patients diagnosed with HPV-related lesions with HPV hr positive typing: particular attention was paid to the last ones, who underwent a higher number of check-ups (at 1, 6, and 12 months).

Another review with conclusions similar to Fiorillo’s, published in 2019 by Orrù et al., reports that outpatient visits to the dentist often represent the “front line” of a diagnostic–therapeutic pathway based on the evaluation of HPV-related lesions in the oral cavity. This means that proper patient management can prevent the degeneration of viral lesions to neoplastic ones, with obvious benefits for the patient [39]. Narrow-band imaging is a tool that has the potential to discern a malignant transformation occurring in some lesions of the oral cavity undergoing long-term follow-up. However, studies are available regarding the use of narrow-band imaging on oral lichen planus or lichenoid lesions, but not HPV-related lesions [40].

As for leukoplakia, data available in the literature indicate that the majority of patients with leukoplakia are over 50 years of age, and only 1% of patients are under 30 [41,42]. Our data are consistent with what has been reported regarding the leukoplakia-age distribution (median age of 65).

In this review, Siracusa et al. (who used the keywords “follow-up”, “oral”, and “leukoplakia”) found that there are no consistent data regarding the follow-up to apply to patients with leukoplakia. Therefore, they believe that it is necessary to standardize a protocol that establishes the frequency and duration of follow-ups and the parameters to consider at each check-up [31]. They also hypothesize that the low % of malignant transformation of leukoplakia that they found (13%) is because few studies analyze extended follow-up over time [31]. However, the % of malignant transformation was not evaluated by our study.

Another literature review that analyzed 24 studies, with a total of 12,703 cases of leukoplakia, reports that check-ups should be every 3 months [32]. Other authors recommend lifelong follow-ups, with a frequency of 6 to 12 months [35]. A recent meta-analysis in 2020 [43], which considered 24 articles for a total of 16,604 cases of leukoplakia, repeats the discrepancy between the follow-up of leukoplakia patients in the various studies, and their analysis seems to be indicative of a necessity for internationally accepted guidelines for the diagnosis and follow-up of leukoplakia cases.

From the data that emerged from this study, patients with leukoplakia had semestral check-ups every 6 months, in line with the suggestions of some studies in the literature.

In a meta-analysis published in 2021 [33], 12 articles were considered, with 397 patients diagnosed with PVL. The median age was 62.34 ± 0.12 years, consistent with the median age of patients in this study, as can be seen from Figure 4. The median follow-up time for the various studies was 79.3 months, with a range of 6–171 months, and 14.6% of cases were HPV positive. Haro and his colleagues report that there is no univocity regarding the treatment, definition of recurrence, and follow-up of patients diagnosed with PVL [33]. They recommend check-ups every 3 to 6 months with photographic documentation to more easily detect clinical changes in the lesion and strongly advise motivating patients to eliminate possible risk factors for the development of oral cancer, such as alcohol and smoking. They conclude that further randomized, controlled, longitudinal, multicenter trials are needed, with a longer follow-up period and a larger patient sample [33].

The mean follow-up of the patients considered by our study was every 4 months for PVL and HPV-positive patients and every 6 months for PVL and HPV-negative patients. The timing of our follow-up (4–6 months) is in line with some evidence in the literature.

An increasing number of epidemiological and molecular studies have demonstrated a strong association between HPV and a large proportion of oral cancers of the oral cavity, tongue, oropharynx, palate, tongue, and tonsils. The prevalence of HPV infection in cancer varies widely based on the geographic region, the HPV DNA detection method used, demographics, the type of clinical specimen used, and the anatomical location of the tumor. Surprisingly, the incidence of HPV-related oral cancer is increasing rapidly (42–70%) in younger populations, mainly in developed countries. High-risk HPV type 16 is the predominant type, accounting for more than 90% of HPV-related oral cancers. Therefore, early detection is currently very important [44].

### 4.2. Recurrence Rate of HPV-Related Oral Lesions

Data in the literature report a rare recurrence rate for HPV-related lesions (papilloma, warts, and condiloma) [45,46,47]. Likewise, a very low recurrence rate was found by our data: only 4.6% of HPV-positive lesions recurred, and on average, it happened within the first 12 months (50% of cases at 12 months, 33.3% at 6 months, and 16.7% at 16 months).

### 4.3. Limits of This Study

The main limitations of this study, as well as the reasons why it was not possible to fill the gap in the literature, are represented by the size of the sample examined and the time interval in which the follow-ups took place. In this study, the follow-ups of 230 patients were evaluated. The number of follow-ups and their frequencies were considered from the time of diagnosis (time 0) until 1 June 2023; therefore, not all cases could have the same follow-up period. The maximum possible follow-up window for 2018 cases was 66 months. However, 2022 patients had a maximum possible follow-up window of 18 months (Table 5).

## 5. Conclusions

The literature is full of studies that analyze HPV-related lesions, leukoplakia, and proliferative verrucous leukoplakia on several levels, but there is a lack of parameters and time indications regarding the management of a follow-up program. Therefore, a standardized protocol that sets follow-up parameters and allows the interface of several professional figures is needed to ensure the most effective therapeutic pathway.

In conclusion, although this study confirms what has already been reported by other authors regarding the criticalities present in the follow-up of these patients, it shows that there was a statistically significant difference in the sample under analysis regarding the follow-up of HPV-positive vs. HPV-negative patients. It also confirms the low frequency of recurrence of HPV-positive oral lesions and clarifies epidemiological aspects in accordance with data available in the literature.

## Figures and Tables

**Table 1:** The database created for the management of patients’ data.

Patient	Age	Sex	Biopsy Date	Histological Exam	Anatomical Site	HPV hr	HPV gr	Follow Up 4 w	2 Months	3 Months	4 Months
Patient 122	61	M	11/01/21	D	b	0	0	1	0	0	0
Patient 123	56	M	12/01/21	B	b	0	0	1	0	0	0
Patient 124	47	F	15/01/21	A	a	0	0	1	0	0	0
Patient 125	62	M	19/01/21	B	d	0	1	1	0	0	0
Patient 126	23	F	04/02/21	B	a	1	1	1	0	0	0
Patient 127	70	M	09/02/21	A	k	0	0	1	0	0	0
Patient 128	64	F	09/02/21	B	e	0	0	1	0	0	0
Patient 129	49	M	09/02/21	B	h	0	1	1	0	0	0
Patient 130	65	F	09/02/21	A	e	0	0	1	0	0	0
Patient 131	30	F	10/02/21	B	g	1	1	1	0	0	0
Patient 132	18	M	23/02/21	B	b	0	1	1	0	0	0
Patient 133	47	F	23/02/21	B	f	1	1	1	0	0	0
Patient 134	48	M	02/03/21	B	i	1	1	1	0	0	0
Patient 135	32	M	09/03/21	C	a	1	0	1	1	0	0
Patient 136	59	F	15/03/21	B	a	0	0	1	0	0	0
Patient 137	39	M	15/03/21	B	c	0	0	1	0	0	0
Patient 138	16	M	16/03/21	B	d	0	1	1	0	0	0

**Table 2:** The four clinical–histological diagnoses and the respective codes with which they were reported in the database.

	Code	Diagnosis
1	A	Clinical–histological picture compatible with traumatic fibroma of the oral mucosa
2	B	Clinical–histological picture compatible with HPV-related lesions ^1^ [Morphological findings compatible with viral cytopathic alterations (HPV) of the oral mucosa: fragments of oral mucosa with epithelial hyperplasia and focal hyperkeratosis and koilocytosis]
3	C	Clinical–histological picture compatible with proliferative verrucous leukoplakia of the oral mucosa (PVL, proliferative verrucous leukoplakia)
4	D	Clinical–histological picture compatible with leukoplakia

^1^ To simplify the statistical analysis, all cases of squamous papilloma, verruca vulgaris, and condyloma acuminatum have been grouped under the heading “HPV-related lesions” (B).

**Table 3:** The anatomical sites of the lesions and the respective code under which they were reported in the database.

	Code	Anatomical Site
1	a	Lateral margin of the tongue
2	b	Retromolar trigone
3	c	Hard palate mucosa
4	d	Soft palate mucosa
5	e	Buccal mucosa
6	f	Vestibular gingiva of the upper maxilla
7	g	Vestibular gingiva of the inferior jaw
8	h	Tongue tip
9	i	Dorsal surface of the tongue
10	j	Ventral surface of the tongue
11	k	Retrocommissure of lower lip
12	l	Lingual gingiva of the upper maxilla
13	m	Upper lip mucosa
14	n	Lower lip mucosa

**Table 4:** Recurrence cases of HPV-related lesions from 2018 to 2022.

	Cases	Age	Sex	Histological Diagnosis	Anatomical Site	HPV hr	HPV gr	Recurrence
1	Patient 22	58	F	B	a	+	+	12 months
2	Patient 23	84	M	B	c	+	+	16 months
3	Patient 168	81	F	B	b	+	+	12 months
4	Patient 176	41	M	B	c	−	+	12 months
5	Patient 179	63	F	B	g	+	+	6 months
6	Patient 194	20	M	B	c	+	+	6 months

**Table 5:** Maximum possible follow-up window.

Year	Maximum Follow-Up Period (Starting from the Date of Diagnosis until 1 June 2023)
2018	66
2019	54
2020	42
2021	30
2022	18

## Data Availability

Data are contained within the article.
